# Predicting the central 10 degrees visual field in glaucoma by applying a deep learning algorithm to optical coherence tomography images

**DOI:** 10.1038/s41598-020-79494-6

**Published:** 2021-01-26

**Authors:** Shotaro Asano, Ryo Asaoka, Hiroshi Murata, Yohei Hashimoto, Atsuya Miki, Kazuhiko Mori, Yoko Ikeda, Takashi Kanamoto, Junkichi Yamagami, Kenji Inoue

**Affiliations:** 1 Department of Ophthalmology, The University of Tokyo, Tokyo, Japan; 2 Department of Ophthalmology, Osaka University Graduate School of Medicine, Osaka, Japan; 3 Department of Ophthalmology, Kyoto Prefectural University of Medicine, Kyoto, Japan; 4 Oike-Ganka Ikeda Clinic, Kyoto, Japan; 5 Hiroshima Memorial Hospital, Hiroshima, Japan; 6 Department of Ophthalmology, Hiroshima Prefectural Hospital, Hiroshima, Japan; 7 Department of Ophthalmology, JR General Hospital, Tokyo, Japan; 8 Inouye Eye Hospital, Tokyo, Japan

**Keywords:** Translational research, Experimental models of disease

## Abstract

We aimed to develop a model to predict visual field (VF) in the central 10 degrees in patients with glaucoma, by training a convolutional neural network (CNN) with optical coherence tomography (OCT) images and adjusting the values with Humphrey Field Analyzer (HFA) 24–2 test. The training dataset included 558 eyes from 312 glaucoma patients and 90 eyes from 46 normal subjects. The testing dataset included 105 eyes from 72 glaucoma patients. All eyes were analyzed by the HFA 10-2 test and OCT; eyes in the testing dataset were additionally analyzed by the HFA 24-2 test. During CNN model training, the total deviation (TD) values of the HFA 10-2 test point were predicted from the combined OCT-measured macular retinal layers’ thicknesses. Then, the predicted TD values were corrected using the TD values of the innermost four points from the HFA 24-2 test. Mean absolute error derived from the CNN models ranged between 9.4 and 9.5 B. These values reduced to 5.5 dB on average, when the data were corrected using the HFA 24-2 test. In conclusion, HFA 10-2 test results can be predicted with a OCT images using a trained CNN model with adjustment using HFA 24-2 test.

## Introduction

Glaucoma is one of the leading causes of blindness worldwide^1^. Here, vision loss is progressive and irreversible and ultimately impacts on the quality-of-life of affected patients^2^. The scope of the visual field (VF) is the principal measurement made when diagnosing and monitoring glaucoma progression. Importantly, measurable structural alterations, such as ganglion cell complex (GCC) loss often precede detectable VF damage^3–6^. The current approach to evaluate GCC thickness is by spectral domain optical coherence tomography (SD-OCT) that measures the thicknesses of the ganglion cell and inner plexiform layers of the GCC^7–14^. Because glaucoma is characterized by irreversible GCC loss, abnormal VF sensitivity might be predicted from the GCC thickness^15^. However, no method has been developed to accurately predict the VF sensitivity from OCT-measured retinal layer thicknesses thus far. Rather, SD-OCT is usually interpreted separately from the VF in daily glaucoma clinics.

A method to predict the VF from GCC thickness might be lacking because the relationship between the two parameters is probably nonlinear; as such, methods such as simple linear regression are likely not be applicable. For instance, the relationship between retinal function and structure [such as retinal nerve fiber layer (RNFL) thickness] is thought to be nonlinear^16^. Hood et al. proposed a nonlinear structure–function model in which the deterioration of visual sensitivity is halted until the RNFL reaches a critical thickness^16^. The researchers also identified a possible ‘floor effect’ in the RNFL thickness^16^. In addition, there is wide inter-individual variation in the retinal structure–function relationship: visual sensitivities differ between individuals for a given RNFL thickness. One possible reason is the original thicknesses of the retinal layers differ before disease development^17,18^.

Glaucomatous retinal damage usually occurs in characteristic patterns^19,20^; thus it is likely that deterioration of the VF also occurs in characteristic patterns. Consequently, previous studies have aimed to interpret the patterns of retinal layer thicknesses measured with OCT using machine learning methods, such as multivariate logistic regression^21^, support vector machine^22^, decision tree classifier^23^, and Random Forests method^24^, to diagnose glaucoma. Likewise, Zhu et al. reported the use of Bayesian framework to predict visual function from peripapillary topography^25^. Such approaches have received renewed interest following the development of the deep learning (DL) methods^26^. DL methods process information via interconnected neurons that are similar to artificial neural networks. However, they also include many ‘hidden layers’ that become computable along with a feature extractor, which transforms raw data into an optimal feature vector that can in turn identify the input data’s patterns^27^. Convolutional Neural Network (CNN) is one such DL method, that might be useful for guiding a glaucoma diagnosis using fundus photographs^28–31^ and/or OCT images^32^.

Currently, the glaucomatous VF change is often analyzed in the clinic using the Humphrey Field Analyzer Central 24–2 test (HFA 24-2, Carl Zeiss Meditec, Dublin, CA); however, the glaucomatous central scotoma can only be accurately assessed by measuring the central 10 degrees, using an analysis such as the Humphrey Field Analyzer Central 10–2 test (HFA 10-2)^5–7^. Performing both tests (or its equivalent) at a sufficient frequency confers a relatively heavy physical and economic burden in the clinic^33,34^. We thus considered that it would be beneficial if the central 10 degrees VF could be accurately predicted from SD-OCT-measured retinal thicknesses and HFA 24-2 test data. In addition, the area of macular scan with SD-OCT mainly corresponds to the area measured with HFA 10-2 VF, and test points with HFA 24-2 VF is out of the correspondence^35^. The clinal importance of central VF cannot be overstated nowadays, because it is more directly associated with the patients’ quality of life^36,37^ and also more directly related to visual acuity than HFA 24-2 VF^38^. Hence, the early use of trabeculectomy is often performed in eyes with the central VF progression, irrespective of the more peripheral areas’ progression status. We thus aimed to develop new CNN models that can predict glaucomatous VF deterioration in the central 10 degrees from SD-OCT and HFA 24-2 VF measurements.

## Methods

### Study approvals and informed consent

This retrospective study was approved by the Research Ethics Committees of the Graduate School of Medicine and Faculty of Medicine at the University of Tokyo, the Osaka University Graduate School of Medicine, the Kyoto Prefectural University of Medicine, the Oike-Ganka Ikeda Clinic, the Hiroshima Memorial Hospital, the Inouye Eye Hospital, and the JR Tokyo General Hospital,Japan. All patients provided written informed consent for their information to be stored in the hospital database and used in this study. This study was performed according to the tenets of the Declaration of Helsinki.

### Study population

#### Training dataset

The testing dataset was obtained between April 2013 and October 2017, recruiting patients from the University of Tokyo Hospital, the Inouye Eye Hospital, the JR Tokyo General Hospital, the Hiroshima Memorial Hospital, the Osaka University Hospital, the Kyoto Prefectural University of Medicine Hospital, and the Oike-Ganka Ikeda Clinic. Here, 648 eyes of 358 subjects, who conducted OCT and HFA 10-2 within 3 months of each other, were selected comprising 90 normal eyes from 46 healthy subjects and 558 eyes with primary open angle glaucoma (POAG) from 312 patients (Table 1). All subjects underwent complete ophthalmic examinations, including biomicroscopy, gonioscopy, intraocular pressure measurement, funduscopy, refraction, best-corrected visual acuity measurement and axial length (AL) measurements, as well as OCT imaging and HFA 10-2 test.

**Table 1:** The demographics and clinical data related to the subjects included in the training dataset.

	Non-glaucomatous eyes	Glaucomatous eyes
Eyes (n)	90	558
Subjects (n)	46	312
Age (years)*	29.9 ± 8.9 [22–78]	60.6 ± 11.2 [29–86]
Laterality (right/left)	45 / 45	274 / 284
MD with HFA 10-2*	−0.3 ± 0.9 [-2.9 to 1.3]	−10.9 ± 9.3 [−33.9 to 2.4]

*The data represent the means ± standard deviation [range]. *MD* mean deviation, *HFA* Humphrey Field Analyzer.

Patients > 18 years-of-age were considered to have primary open-angle glaucoma (POAG) if they exhibited the following: (1) typical glaucomatous changes in the optic nerve head, such as a rim notch with a rim width/disc diameter ratio of ≤ 0.1, a vertical cup-to-disc ratio of > 0.7, and/or a retinal nerve fiber layer defect with its edge at the optic nerve head margin greater than a major retinal vessel, diverging in an arcuate or wedge shape; (2) grade 3 or 4 gonioscopically wide open angles based on the Shaffer classification; (3) visual acuity ≤ 1.0 LogMAR; and (4) a refractive error <  + 3.0 diopter. Exclusion criterion were (1) age < 18 years; (2) possible secondary ocular hypertension in either eye; and/or (3) the presence of any other systemic or ocular disorders.

Inclusion criteria for non-glaucomatous patients were: (1) no abnormal findings except for clinically insignificant senile cataract on biomicroscopy, gonioscopy, and funduscopy; (2) no history of ocular disease that could affect the results of OCT examinations, such as diabetic retinopathy or age-related macular degeneration; (3) aged between 18 and 80 years old; (4) normal VF test results according to the Anderson-Patella criteria^39^; and (5) a refractive error <  + 3.0 diopter. Eyes which had the measurement of HFA 10-2 test and HFA 24-2 test within 3 months from the OCT imaging were used as testing data, otherwise used as training data (see below).

#### Testing dataset

The testing dataset was obtained between April 2013 and October 2017, recruiting patients from the University of Tokyo Hospital, the Inouye Eye Hospital, the JR Tokyo General Hospital, the Hiroshima Memorial Hospital, the Osaka University Hospital, the Kyoto Prefectural University of Medicine Hospital, and the Oike-Ganka Ikeda Clinic. The dataset comprised 105 eyes from 72 POAG patients (Table 2). The inclusion and exclusion criterion were the same as those for the training dataset, and the same measurements were taken. All of the subjects underwent HFA 10-2 test and HFA 24-2 test within 3 months of undergoing OCT.

**Table 2:** The demographics and clinical data related to the subjects included in the testing dataset.

	Glaucomatous eyes
Eyes (n)	105
Subjects (n)	72
Age (years)*	60.6 ± 12.8 [18 to 90]
Laterality (right/left)	53 / 52
BCVA, LogMAR	−0.01 ± 0.17 [−0.18 to 1]
AL (mm)	25.3 ± 1.6 [21.8 to 28.8]
MD with HFA 10-2*	−10.5 ± 9.1 [−34.6 to 1.6]

*The data represent the means ± standard deviation. *BCVA* best corrected visual acuity, *MD* mean deviation, *HFA* Humphrey Field Analyzer.

#### VF testing

The scope of the VF was analyzed by HFA 10-2 test, according to the Swedish Interactive Threshold Algorithm (SITA) Standard strategy; the total deviation (TD) value of the 68 observation points was determined. Near refractive correction was used as necessary.

In the testing dataset, the HFA 24-2 test was performed within 3 months of the HFA 10-2 test. All of the participants had had previously undergone a VF examination and unreliable VFs — defined as fixation losses > 33%, false-positive responses > 33%, or false-negative > 33% — were excluded.

#### SD-OCT measurement

SD-OCT data were obtained using an RS 3000 OCT system (Nidek Co ltd., Aichi, Japan), and AL measurements were generated using an OA-2000 optical biometer (TOMEY, Aichi, Japan). All SD-OCT measurements were carried out after pupil dilation with 1% tropicamide and OCT was performed using a laser scan protocol. Any data contaminated by eye movements, involuntary blinking and/or saccade were excluded. Imaging data with a quality factor < 7 were also excluded, as recommended by the manufacturer.

The fovea was automatically identified as the pixel closest to the fixation point containing with thinnest retinal thickness. A square imaging area (9.0 × 9.0 mm) was then centered on the fovea. Using software supplied by the manufacturer, the thicknesses of the i) RNFL, ii) GCC, and iii) outer segment (OS) + retinal pigment epithelium (RPE) were exported from the square imaging area as a digital matrix (512 × 128 pixels). Including the OS + RPE thickness improves the structure–function relationship between the VF sensitivity and the retinal thickness^40,41^, thus the OS + RPE thicknesses were used in this study. Prior to CNN training, the thickness of each layer determined by OCT was resized to 224 × 224 pixels using a bilinear interpolating method^42^ following previous study to satisfy the CNN models’ input data size^43^.

### CNN models

#### Visual Geometry Group (VGG)

The VGG is a DL, CNN model using very small (3 × 3 pixels) convolution filters^44,45^. In this study, a VGG model^44^ with 19 hidden layers (VGG19) was used. The converted depths of each layer measured by OCT were inputted into the VGG. The VGG output was defined as a numerical vector of predicted visual sensitivity at each location.

#### Deep Residual Learning for Image Recognition (ResNet)

ResNet is a recently proposed DL, CNN algorithm^46^ that overcomes issues related to a vanishing gradient or gradient divergence via its unique structure of ‘identity shortcut connections’ that skip one or more layers. In this study, Resnet152 (which contains 152 hidden layers) was used. The converted depths of each layer and the output format were as described for VGG.

#### Model training

The VGG and ResNet models were trained to predict each of the 68 TD values that would derive from the HFA 10-2 test using macular GCC, RNFL, and OS + RPE thicknesses determined from the training dataset. These CNN models were constructed with the 224 × 224 pixels RNFL thickness (in the first channel), the GCC thickness (in the second channel) and the OS + RPE thickness (in the third channel). DL usually requires a large training dataset, which often cannot be prepared in the clinical setting. Transfer learning is a method to overcome this problem by pre-training DL models using a heterogeneous dataset^26,47^. Traditional machine learning techniques try to learn each task from scratch, while transfer learning techniques try to transfer the knowledge from some previous tasks to a target task when the latter has fewer high-quality training data^48^. Hence, the diagnostic ability of a DL model can be improved by conducting a preliminary training using a very different and large-sized dataset. Here, the pre-trained VGG19^44^ and Resnet152^43^ neural networks trained using the ImageNet data (http://www.image-net.org/), which consists of 1,000 categories.

### Statistical analyses

The prediction performances of the VGG and ResNet models were validated using the testing dataset, and were evaluated using the mean absolute error (MAE_VGG_ and MAE_Resnet_) approach^49^, as follows:

$${\text{MAE}} = \frac{{\mathop \sum \nolimits_{i = 1}^{j} {\text{|predicted}}\,{\text{visual}}\,{\text{sensitivity}}\,{\text{of}}\,{\text{the}}\,i{\text{th}}\,{\text{point}} - {\text{actual}}\,{\text{visual}}\,{\text{sensitivity}}\,{\text{of}}\,{\text{the}}\,i{\text{th}}\,{\text{point|}}}}{j},$$

where j = the number of the predicted 68 test points.

**Figure 1:**
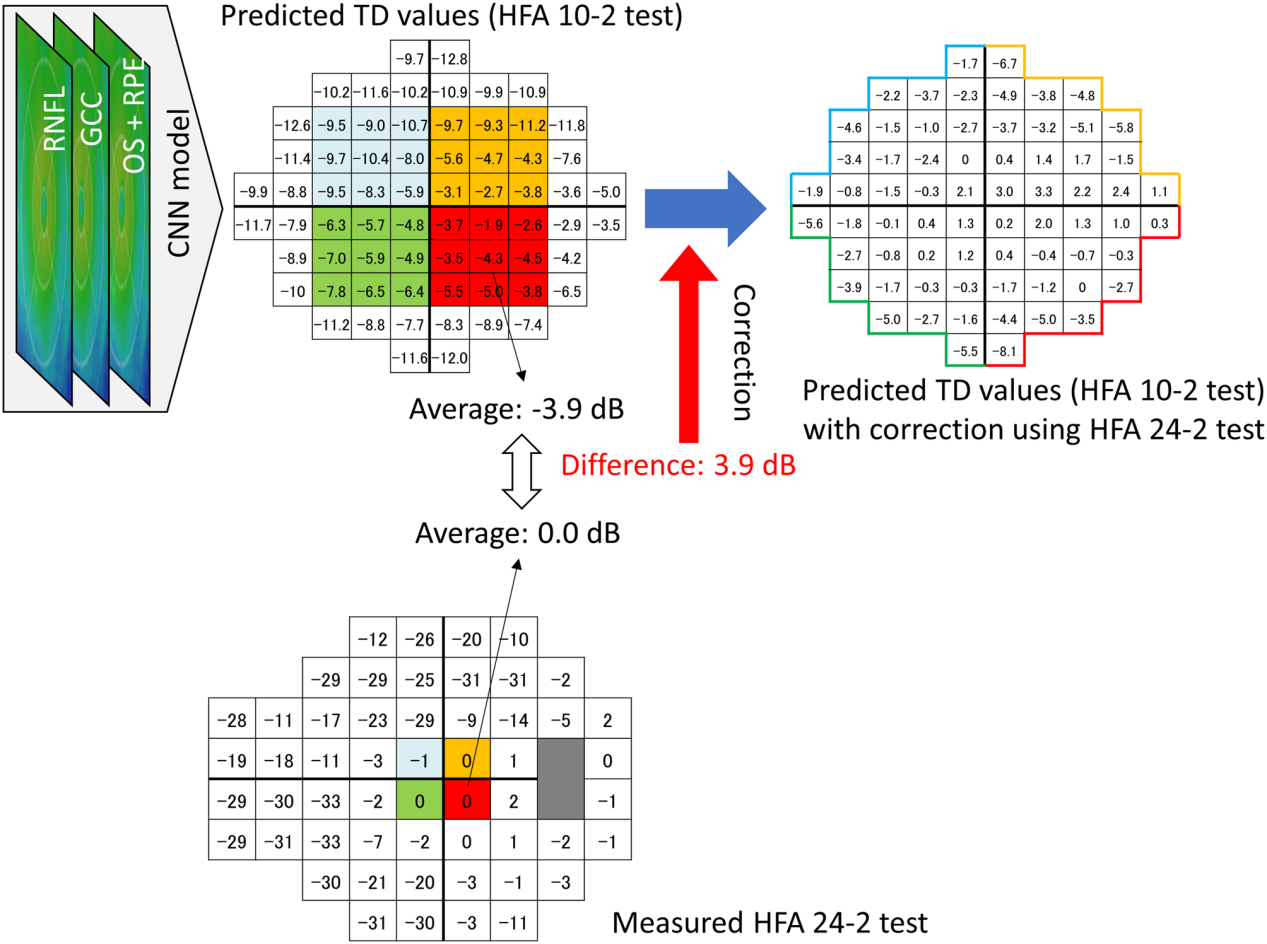
The CNN model. A CNN model was used to predict TD values in the central 10 degrees of the visual field (corresponding to HFA 10-2 test data). The mean TD values within the central 6 degrees at each quadrant (36 and four test points for the HFA 10-2 and HFA 24-2 tests, respectively) were calculated. The corresponding test grids in the inferonasal quadrant are shown. The differences between these values were calculated, and the predicted TD values were adjusted as per the calculated differences in each sector. *CNN* convolutional neural network, *GCC* ganglion cell complex, *HFA* Humphrey Field Analyzer, *OS* outer segment, *RNFL* retinal nerve fiber layer, *RPE* retinal pigment epithelium, *TD* total deviation.

The predicted TD values for the 68 test points by both models (MAE_VGG_, and MAE_ResNet_) were corrected using the TD values of the inner-most four test points derived from the HFA 24-2 test (Fig. 1) that overlap with the HFA 10-2 test points. Specifically, the mean TD values within the central 6 degrees at each quadrant (36 and four test points with HFA 10-2 and HFA 24-2, respectively) were determined, and the differences between these values were calculated. Then, the TD values of the predicted HFA 10-2 test data were corrected using this difference (MAE_VGG_adjust_, and MAE_ResNet_adjust_). As a baseline method, the MAE value was calculated by applying the linear interpolation method using the innermost four test points’ results in HFA 24-2 test (MAE_Interpolation_). The *R*^2^ (a square of a correlation coefficient) was calculated between the measured TD values and MAE_VGG_, MAE_VGG_adjust_, MAE_ResNet_, MAE_ResNet_adjust_, and MAE_Interpolation_.

Subsequently, the MAE of mean TD (mTD) values between measured and predicted HFA 10-2 tests (adjusted VGG and ResNet algorithm) were calculated. The associations between the MAE values and the reliability indices of VF (fixation loss, false negative rate, and false positive rate) were also evaluated.

All statistical analyses were carried out using the statistical programming language Python (version 3.6.4; Python Software Foundation, https://www.python.org/). P values in multiple comparisons were corrected using the Bonferroni’s multiple comparison method.

## Results

**Figure 2:**
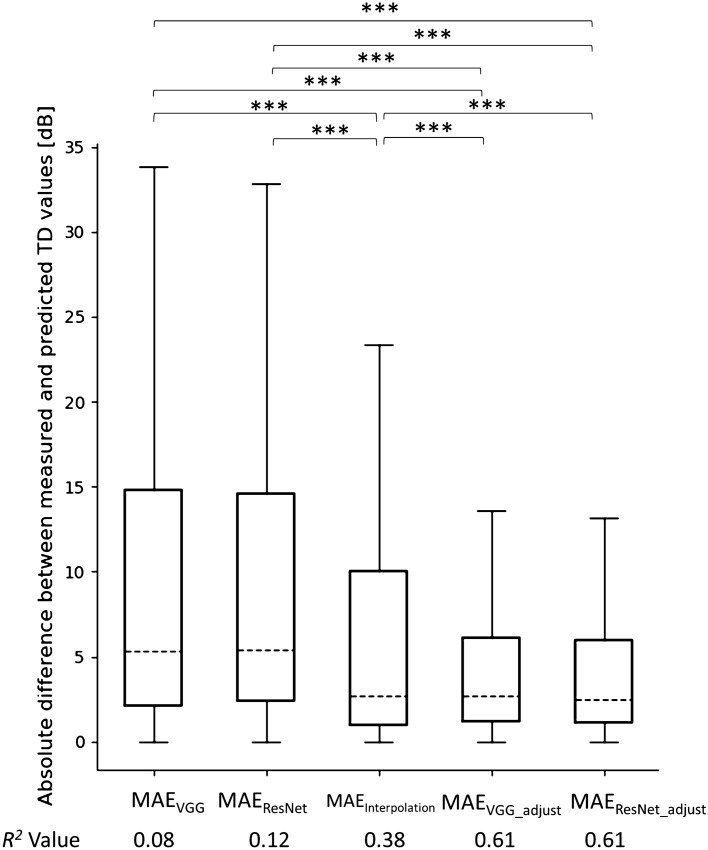
The absolute difference between the measured and predicted TD values. The boxplot shows, 75% quartile + 1.5 × (75% quartile – 25% quartile), 75% quartile, median, 25% quartile, and 25% quartile—1.5 × (75% quartile – 25% quartile) values. ***, p < 0.001; MAE, mean absolute error; TD, total deviation.

We first measured the MAE of the predicted TD values. We found that the unadjusted MAE_VGG_, and MAE_ResNet_ values were 9.5 ± 9.4 dB [mean ± standard deviation (SD)] and 9.4 ± 9.3 dB, respectively (Fig. 2). There was no statistically significant difference between MAE_ResNet_ and MAE_VGG_ (*p* = 1.0, Mann–Whitney U test with Bonferroni’s adjustment for multiple comparisons). We then corrected the predicted TD values using the HFA 24-2 data. Here, the MAE_VGG_adjust_ value was 5.4 ± 7.0 dB, which was significantly smaller than the MAE_VGG_ value (*p* < 0.0001). Similarly, the MAE_ResNet_adjust_ value was 5.3 ± 7.0 dB, which was significantly smaller than the MAE_ResNet_ value (Fig. 2, *p* < 0.0001). MAE_Interpolation_ was 7.4 ± 9.1 dB, which was significantly greater compared with MAE_VGG_adjust_ and MAE_ResNet_adjust_ (*p* < 0.0001). The *R*^2^ values between the adjusted TD values derived from the VGG, ResNet, linear interpolation, adjusted VGG, and adjusted ResNet models, and the actual TD values were 0.08, 0.12, 0.38, 0.61 and 0.61, respectively (Fig. 2).

The MAEs of mTD between measured and predicted HFA 10-2 test were 2.50 and 2.53 dB with adjusted VGG and ResNet models, respectively. These MAE values were significantly positive correlated with false negative rate of HFA 10-2 test (both p < 0.0001, Pearson’s correlation test), but not with fixation loss (p = 0.41 and 0.35) and false positive (p = 0.63 and 0.59).

**Figure 3:**
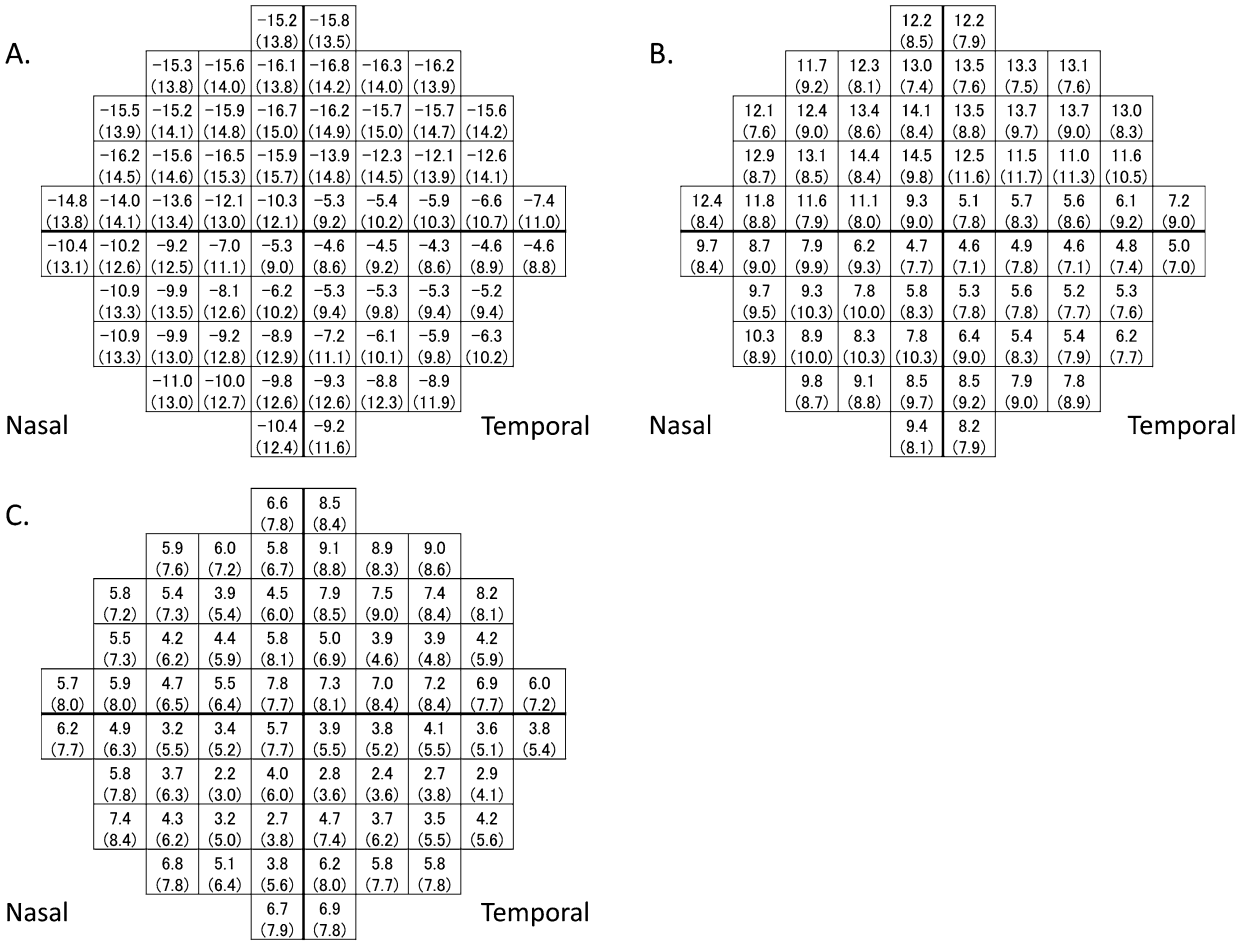
The predicted TD values using ResNet model. A. The mean (standard deviation) TD values at each test point with the actual HFA 10-2 test. B. The MAE (standard deviation) of the predicted TD values using the ResNet model without correction with HFA 24-2 test data at each test point. C. The MAE (standard deviation) of the predicted TD values using the ResNet model, with correction using the HFA 24-2 data at each test point. The schematic is representative of the right eye. *HFA* Humphrey Field Analyzer, *MAE* mean absolute error, *TD* total deviation.

Figure 3A shows the mean TD values at each test point with the actual HFA 10-2 test. The MAEs of predicted TD values using ResNet model with or without HFA 24-2 based correction at each observation point were shown in Fig. 3B,C, respectively. With ResNet, MAE value was significantly smaller with HFA 24-2 base correction than without it at 49 out of 68 (72.1%) test points (Mann–Whitney U test with Holm’s adjustment for multiple comparisons). In general, we observed small prediction error values in the inferior-temporal area.

**Figure 4:**
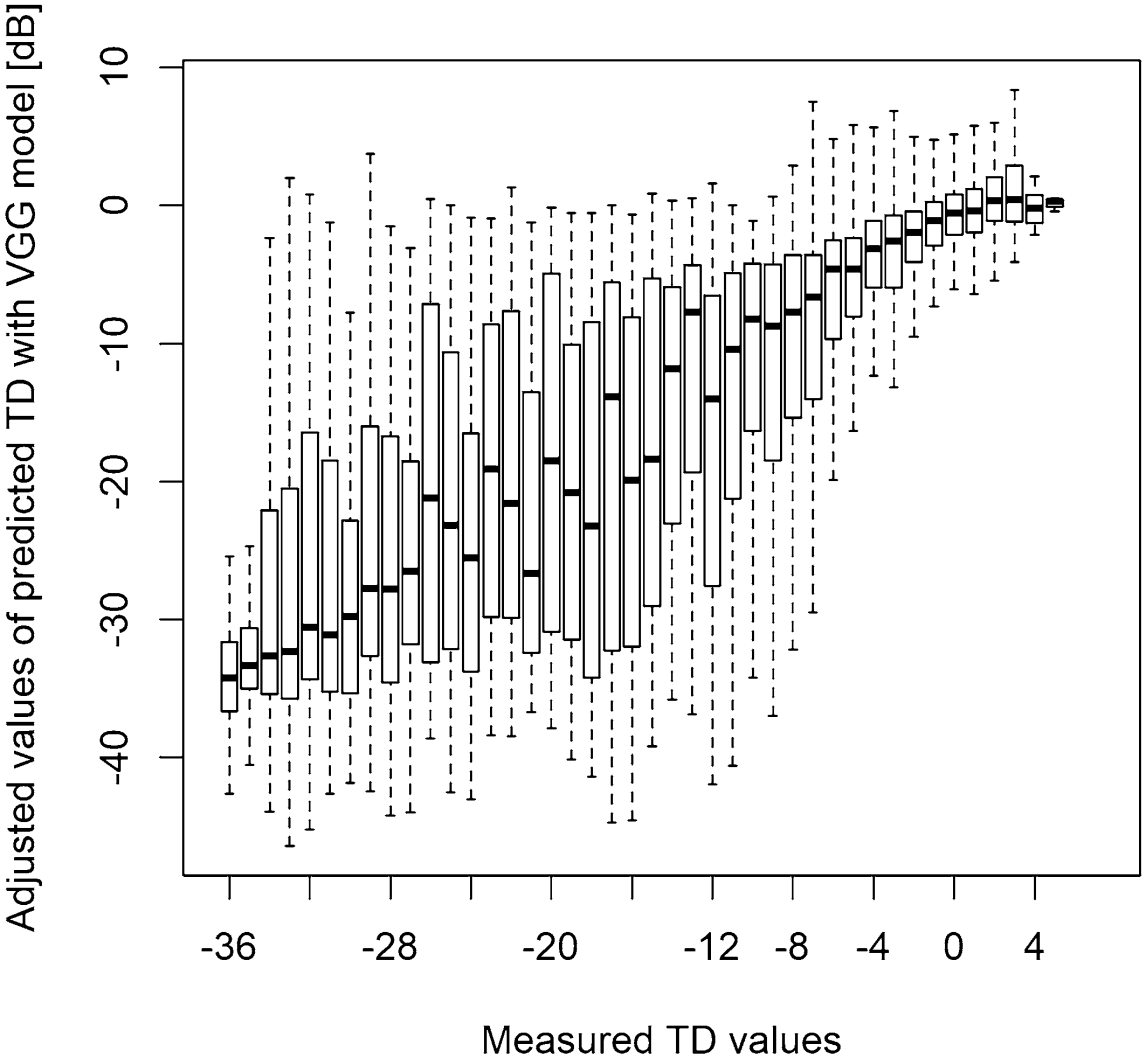
The relationship between the actual TD values and the predicted TD values using the VGG model adjusted with the measured TD values corresponding to the innermost four points of the HFA 24-2 test. *HFA* Humphrey Field Analyzer, *TD* total deviation.

**Figure 5:**
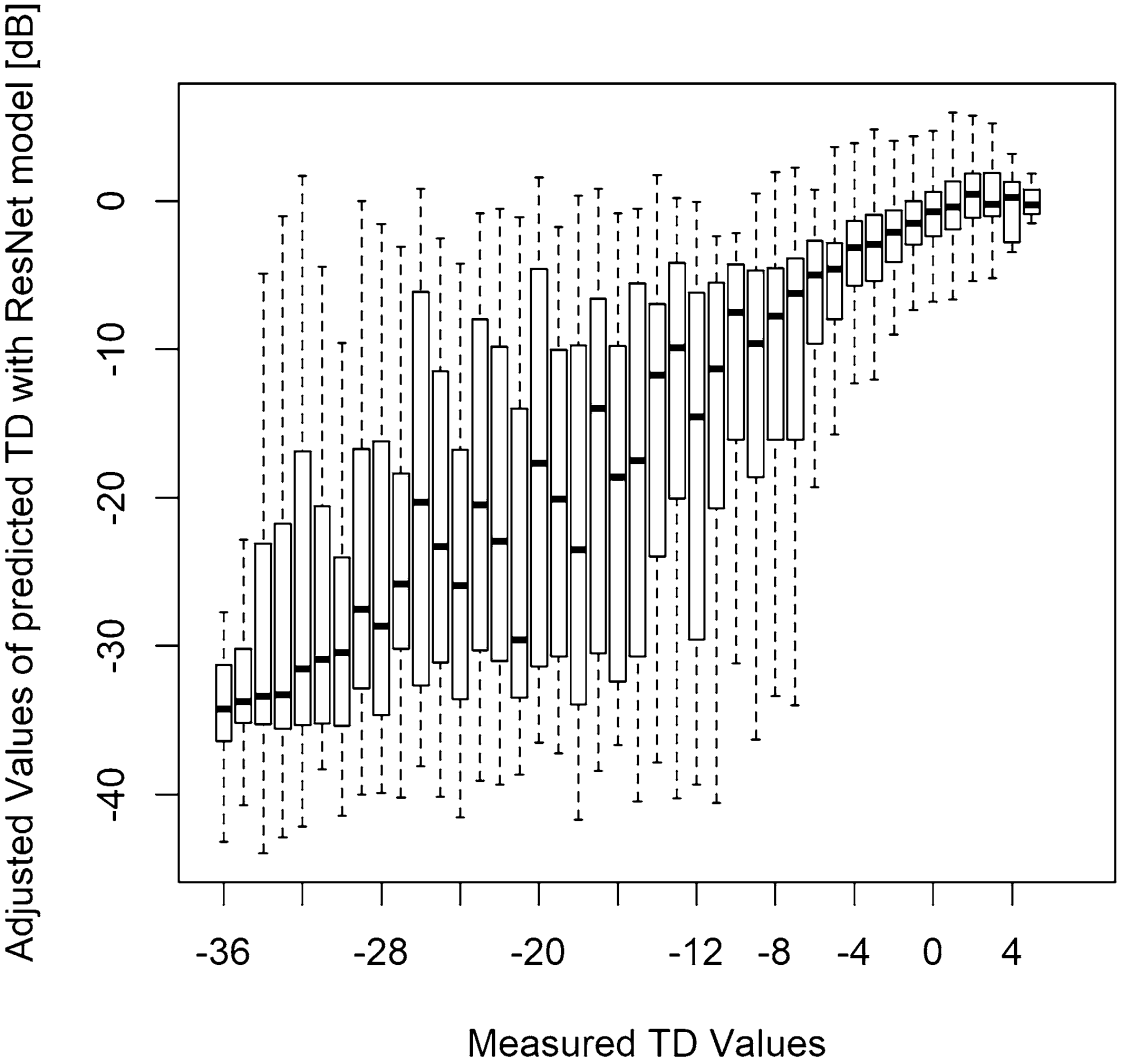
The relationship between the actual TD values and the predicted TD values using the ResNet model adjusted with the measured TD values corresponding to the innermost four points of the HFA 24-2 test. *HFA* Humphrey Field Analyzer, *TD* total deviation.

Finally, we determined the relationship between the measured TD values and the MAE_VGG_adjust_ and MAE_ResNet_adjust_ values following a previous study^50^ (Figs. 4, 5). In general, we found that the prediction error was tight where the measured TD values were either high or low.

## Discussion

In the current study, we developed two CNN models to predict the central 10 degrees VF from OCT-measured retinal thicknesses (macular GCC, RNFL, and OS + RPE thickness). Both the ResNet and VGG algorithms conferred a VF prediction accuracy (in terms of MAE values) of ~ 10 dB. These prediction performances were significantly improved by adjusting the models with the measured TD values from the innermost four tests points generated from the HFA 24-2 test. These data suggest that VF sensitivity within central 10 degrees can be predicted in combination with OCT images and HFA 24-2 test results.

The ResNet algorithm has been reported to yield better prediction/classification performances than the VGG algorithm^43^, because of the higher depth in ResNet^46^. We previously reported that the ResNet algorithm (with 18 layers) enabled a better diagnostic accuracy for glaucoma than the VGG algorithm (with 16 layers) when using fundus photographs as the input^31^. In the present study, although the MAE values with ResNet algorithm were smaller than those with VGG algorithm, there were no statistically significant differences between two algorithms. when using OCT-measured retinal thicknesses as the input.

A previous study by Sugisaki et al. showed that the predicted HFA 10-2 TD values from HFA 24-2 measurements using support vector regression conferred a prediction error of 4.0 ± 1.5 dB^51^. The MAEs derived in our study (average: 9.4 dB and 9.5 dB for MAE_ResNet_ and MAE_VGG_, respectively) are relatively greater than that of the previous study. Although we significantly improved on these prediction errors by using the innermost four tests points from the HFA 24-2 test (average: 5.4 and 5.3 dB for MAE_VGG_adjust_ and MAE_ResNet_adjust_, respectively), these values were still considerably larger than that reported by Sugisaki et al.^51^ This difference could be attributed to differences in the study cohort. Sugisaki et al. included only eyes from patients with advanced glaucoma (mean deviation with HFA 24-2 test < -20 dB), whereas our study included a much range of wider disease stages (range: -34.5 to 1.6) in the testing dataset. Indeed, the prediction errors generated by the two models were smaller when the disease stage was advanced (Figs. 4, 5) because the VF sensitivity approached floor.

The MAE of the TD values at the infero-temporal area were the smallest compared to all other regions (*i.e.* supra-temporal, supra-nasal, infero-nasal, Fig. 3C). As reported by Weber et al., there is a “central isle” in the VF that remains preserved until the late stages of glaucoma^52^; this area corresponds to the infero-temporal area. Consistently, Hood et al. reported that this area corresponds to the maculo-papillary bundle and as a result, it is the least vulnerable region to glaucomatous damage^53^. Previous studies have also reported that OCT is more useful in the early rather than late stages of glaucoma^16,54^. Together, these findings imply that predicting the VF sensitivity in the infero-temporal area is more precise than those in predicting the sensitivity in other regions, because of VF sensitivity preservation. We also found that the variation of the TD values was relatively smaller in this area compared to other areas of the VF, as shown by the small standard deviation values in general. This is another factor that would contribute to the better prediction accuracy in this area.

In the current study, we corrected the predicted HFA 10-2 TD values using the measured HFA 24-2 TD values of the innermost four test points. This simple adjustment accomplished better prediction by more than 40% (Fig. 2). Similarly, we previously reported that when analyzing the MD trend alongside the HFA 10-2 test data, it was advantageous to use predicted MD values for the HFA 10-2 test using HFA 24-2 test data when only a limited number of HFA 10-2 test data are available^55^. Our current results are in agreement, suggesting that a similar approach of using HFA 24-2 test data is also useful when predicting HFA 10-2 test data. Christopher et al. described a CNN method (ResNet50) to predict sectoral pattern deviation in HFA 24-2 test points from the OCT-measured optic nerve head RNFL thickness map, an RNFL en face image, and a confocal laser ophthalmoscopy image^49^. The model was trained using 9,765 OCT images, and the resulting *R*^2^ value between the predicted and the measured pattern deviation values was between 0.08 and 0.15 in the central area. In the current study, we predicted the TD values in a point-wise manner, which is usually more challenging than predicting the values in a manner of global index, but achieved much higher *R*^2^ values (0.61 and 0.61 for the VGG and ResNet models, respectively). When we calculated the mean TD value in the central 10 degrees according to the method used by Christopher et al.^49^, we achieved an even higher *R*^2^ value (0.90, data not shown). These data suggest that foveal OCT images can also be used in estimating VF sensitivity in central 10 degrees, in addition to optic nerve head OCT images.

Previous studies have suggested that the central VF cannot be accurately assessed without using a specified program to determine the VF in the central 10 degrees, such as the HFA 10-2 test^53,56^. However, in clinical settings, performing the HFA 24-2 test with a sufficient frequency already imposes a time and financial burden^33,34^; performing an HFA 10-2 test would only add to this burden. Our data suggest that VF sensitivity in central 10 degrees can be predicted using OCT images in combination with HFA 24-2 test. We thus suspect, therefore, that predicting the central HFA 10-2 test data from OCT-measured retinal thicknesses might be a possible clinical approach.

There are couple of limitations in our study. Following the previous study^43^, we resized the OCT images to 224 × 224 pixels; however, this data processing caused the loss of information, which might have lessen the prediction accuracy. Next, we could only predict the TD values in the central 10 degrees, whereas the VF over a wider area (e.g. 24 degrees) is usually determined in the clinical setting using a test such as the HFA 24-2 test. As a macular OCT scan does not cover such a wide area, the current approach cannot be used to directly predict HFA 24-2 data. Nonetheless, a detailed measurement of the central 10 degrees is essential for diagnosing and monitoring glaucoma^5–7^. In the current study, we constructed a model to predict HFA 10-2 test values using SD-OCT measurements. The prediction accuracy was significantly improved when we corrected the predicted TD values using HFA 24-2 test data. Nonetheless, a careful consideration is still needed when applying this method at the clinical settings. For instance, considerably large standard deviation values were still large even with MAE_ResNet_adjust_. Of note, the prediction accuracy was significantly increased when the reliability (false negative rate) is increased, which suggest that the prediction error was, at least partially, derived from inaccurate VF measurement. DL is not fully matured yet, and studies such as this may represent steps along the way to achieving clinical utility. Nonetheless, it is important to keep in mind the future clinical goal and the requirements to reach it. Going forward, further work is needed to achieve more precise prediction accuracy of glaucomatous VF sensitivity in the central 10 degrees for the clinical application. We then anticipate that the larger training dataset might accomplish better prediction accuracy. We hope that improving HFA 10-2 prediction will result in better management of glaucoma patients.
